# The relationship between low prolactin and type 2 diabetes

**DOI:** 10.1007/s11154-024-09886-w

**Published:** 2024-05-18

**Authors:** Gie Ken-Dror, David Fluck, Michael E. J. Lean, Felipe F. Casanueva, Thang Sieu Han

**Affiliations:** 1 Institute of Cardiovascular Research, Royal Holloway, University of London, Egham, Surrey TW20 0EX UK; 2 Department of Cardiology, Ashford and St Peter’s NHS Foundation Trust, Guildford Road, Chertsey, Surrey KT16 0PZ UK; 3 Department of Human Nutrition, University of Glasgow, Glasgow, UK; 4 Department of Medicine, CIBER de Fisiopatología Obesidad y Nutricion, Instituto Salud Carlos III, SCB06/03, Santiago de Compostela University, Complejo Hospitalario Universitario de Santiago (IDIS), Santiago de Compostela, Spain; 5 Department of Endocrinology, Ashford and St Peter’s NHS Foundation Trust, Guildford Road, Chertsey, Surrey KT16 0PZ UK

**Keywords:** Hypoprolactinemia, Meta-analysis, Sex-specific analysis

## Abstract

Prolactin (PRL) is secreted throughout life in men and women. At elevated levels, its physiological role in pregnancy and lactation, and pathological effects, are well known. However clinical implications of low circulating PRL are not well established. We conducted a meta-analysis to examine the relationship between low PRL levels and type 2 diabetes. Five papers included cross-sectional studies comprising 8,720 men (mean age range 51.4–60 years) and 3,429 women (49.5–61.6 years), and four papers included cohort studies comprising 2,948 men (52.1–60.0 years) and 3,203 women (49.2–60.1 years). Individuals with pregnancy, lactation and hyperprolactinemia, drugs known to alter circulating PRL levels, or pituitary diseases had been excluded. Although most studies used quartiles to categorize PRL groups for analysis, PRL cut-off values (all measured by chemiluminescence immunoassay) were variably defined between studies: the lowest PRL quartiles ranged from 3.6 ng/ml to 7.2 ng/ml in men and between 4.5 ng/ml to 8 ng/ml in women; and the highest PRL quartiles ranged from 6.9 ng/ml to 13 ng/ml in men and 9.6 ng/ml to 15.8 ng/ml in women. Type 2 diabetes was defined variably using self-reported physician’s diagnosis, fasting blood glucose, oral glucose tolerance test or glycated hemoglobin (HbA_1C_). In cross-sectional studies, compared to individuals in the highest PRL groups (reference), those in the lowest PRL groups had greater risk of type 2 diabetes both in men: odds ratio (OR) and 95% confidence interval = 1.86 (1.56–2.22) and in women: OR = 2.15 (1.63–2.85). In cohort studies, women showed a significant association between low PRL and type 2 diabetes: OR = 1.52 (1.02–2.28) but not men: OR = 1.44 (0.46–4.57). Relatively low heterogeneity was observed (*I*^2^ = 25–38.4%) for cross-sectional studies, but higher for cohort studies (*I*^2^ = 52.8–79.7%). In conclusion, low PRL is associated with type 2 diabetes, but discrepancy between men and women in the relationship within cohort studies requires further research.

## Introduction

Within physiological ranges, prolactin (PRL) has multiple biological actions on health [1–3]. Above these ranges, high serum PRL (hyperprolactinemia), driven by hypothalamo-pituitary diseases and by many pharmacological agents, has an adverse impact on bodily functions including a disruption to the reproductive system [4, 5]. By contrast to the well-established area of research on hyperprolactinemia, there is a paucity of studies on the effects of low PRL levels on health outcomes. Evidence from existing literature suggests an association between low PRL and an increased prevalence of type 2 diabetes in both sexes, mostly from cross-sectional studies [6–9]. The few prospective studies carried out also show a low baseline PRL level was related to greater future risk of type 2 diabetes in women [7, 10–12], but this relationship was not demonstratable in men [7, 11], meta-analysed by de Castro et al. [13]. In a new study of over 3,000 men aged 40–86 years published in this issue, we have shown that within the physiological range of PRL (up to 34.9 ng/ml), men with a low PRL level (< 3 ng/ml) at baseline had greater risk of developing type 2 diabetes by over five-fold compared to those with a high PRL level (≥ 5 ng/ml) [14]. With the additional studies published more recently, we have here generated an updated meta-analysis to examine the relationship between low levels of PRL and type 2 diabetes.

## Methods

### Search strategy and data extraction

A protocol for this study was developed prospectively and registered in PROSPERO [https://www.crd.york.ac.uk/prospero/] (submitted 30 March 2024, ID: 530,567, and registered 04 April 2024, registration number: CRD42024530567). This study followed guidelines from the Cochrane and PRISMA recommendations for conducting meta-analyses [15, 16]. After the proposal of the concept of the study, two investigators (GK-D and TSH) carried out independently a literature search and extracted data from papers in MEDLINE, Google Scholar and the Cochrane Database of Systematic Reviews up to May 2024. Title and abstract searches were conducted in all databases using the following search terms: prolactin, PRL, type 2 diabetes and T2DM. No filters for language or data were applied and the Boolean operators “AND” and “OR” were used to combine search terms. Relevant studies were searched from references within the identified papers. Before creating a final database for meta-analysis from data extracted, the search results from the pair of investigators were compared and any disagreements were resolved by consensus.

### Selection criteria

All studies examining the relationship between PRL levels and type 2 diabetes were included in adults (≥ 18 years) irrespective of race, comorbidities, or duration of follow-up. Those that fit the inclusion criteria were cross-sectional or cohort studies, and studies specifically compared the risk of diabetes between low PRL against high PRL categories (*e.g.* quartiles) using cutoffs as presented in the primary studies. Studies were excluded if they presented both sexes together or measurement of PRL during pregnancy or early stages of postpartum which would likely to include lactating women.

### Risk of bias assessment

The risk of bias was assessed independently by two investigators (GK-D and TSH) using the Joanna Briggs Institute Critical Appraisal Checklist for Cohort Studies and Case–Control Studies [17]. The risk of bias for cross sectional and cohort studies was assessed by 10 and 11 questions respectively. The response to the question was “yes”, “no”, “unclear” or “not applicable”. The total number of “yesses” was expressed in percentages: (the number of yesses divided by 10,100%) for cross sectional studies and (the number of yesses divided by 11,100%) for cohort studies. The risk of bias for each study was graded as high, moderate or low if the percentage of yesses were < 50%, 50–69% or ≥ 70% respectively.

### Statistical analysis

Meta-analyses were carried out using Review Manager (RevMan, Version 5.3. Copenhagen: The Nordic Cochrane Centre, the Cochrane Collaboration, 2014). Odds ratios (OR) and 95% confidence intervals were used on the original measurement scale to determine the size of the effect of low PRL levels on type 2 diabetes. Pooled estimates of outcomes were obtained via the DerSimonian-Laird method using a random effects model [18]. Statistical significance threshold was accepted as *P* < 0.05, and heterogeneity of study results were assessed by the *I*^2^ statistic [19].

## Results

### Characteristics

**Fig. 1:**
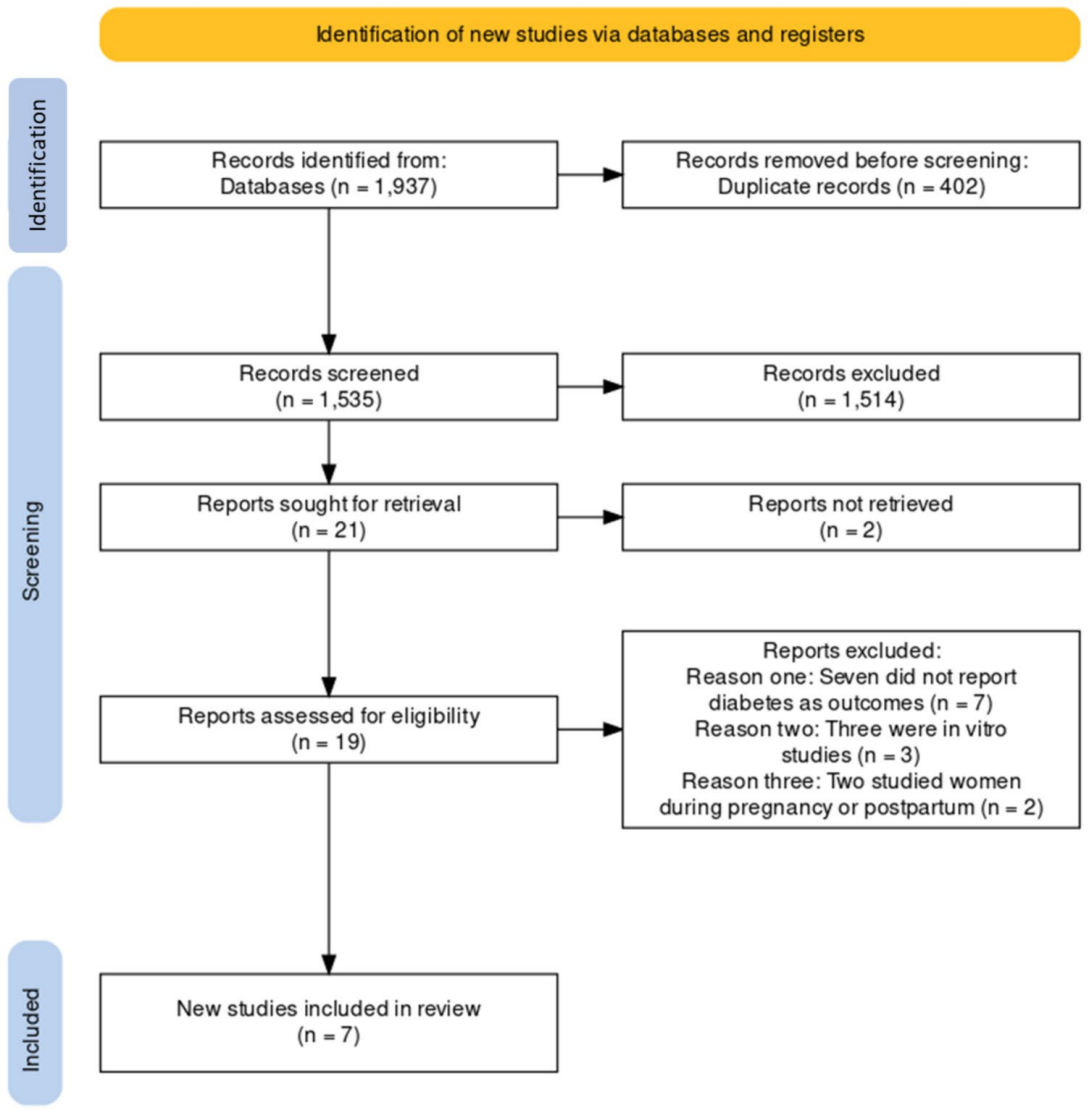
Flow chart of literature search and study selection process

A PRISMA 2020-compliant flow diagram was created to describe the results obtained from literature search [20]. A total of 1,937 titles were initially identified, of which 402 were found to be not relevant. The remaining 1,535 articles were screened and 1,514 were excluded because no original data were presented, leaving twenty-one papers being sought for retrieval, of which two were excluded. Further review of the remaining 19 full texts, to check for eligibility against predefined criteria, found 12 articles to be ineligible because there were incomplete data, in vitro studies or study of pregnant or lactating women. The final seven papers met inclusion criteria for meta-analysis (Fig. 1). These papers included two from China [8, 11] and one each from Italy [6], Germany [7], India [9], the US [10], and eight centres from the European Union (Florence, Italy; Leuven, Belgium; Malmö, Sweden; Manchester, UK; Santiago de Compostela, Spain, Łódź, Poland; Szeged, Hungary; Tartu, Estonia) [14]. All papers were written in English and published between 2009 and 2024. Three studies were cross-sectional only [6, 8, 9], two were cohort only [10, 11], with two contained both study designs [7, 14] (Table 1).

**Table 1:** Prolactin values in different studies. Conversion factor ng/ml = mIU × 0.047

		**Men**			**Women**		
	**Follow-up duration (yrs)**	**Age (yr)**	**Low PRL levels (ng/ml)**	**High PRL levels (ng/ml)**^**a**^	**Age (yr)**	**Low PRL levels (ng/ml)**	**High PRL levels (ng/ml)**^**a**^
**Cross-sectional**							
Corona et al., 2009 [6]	–	Mean:52 (SD 12.9)	Q1: < 5	Q4: ≥ 11.1	–	–	–
Balbach et al., 2013 [7]	–	Median: 52.1 (IQR: 37.4–65.3)	Q1: < 3.6^b^	Q4: > 6.9^b^	Median: 49.5 (IQR: 36–62.3)	Q1: < 4.5^b^	Q4: ≥ 9.6^b^
Wang et al., 2013 [8]	–	Median: 60 (IQR: 54–67.8)	Q1: < 6.4	Q4: ≥ 10.6	Median: 60.5 (IQR: 55.3–70.1)	Q1: < 6.7	Q4: ≥ 11.5
Chahar et al., 2017 [9]	–	Mean: 52.3–53.5 (SD: 5.4–6.4)	Q1: < 7.2	Q4: ≥ 12.6	Mean: 55.8–56.1 (SD: 4–5.5) (all postmenopausal)	Q1: < 7.6	Q4: ≥ 13.4
Han et al., 2024 [14]	–	Mean: 60 Range: 40–86	5th centile: < 3.0	75th centile: ≥ 5.0	–	–	–
**Cohort studies**							
Balbach et al., 2013 [7]	5	Median: 52.1 (IQR: 37.4–65.3)	Q1: < 3.6^b^	Q4: > 6.9^b^	Median: 49.5 (IQR: 36–62.3)	Q1: < 4.5^b^	Q4: ≥ 9.6^b^
Wang et al., 2016 [11]	3.7	Mean: 57.5–63.6 (SD: 7.9–9.9)	Q1: < 5.7	Q4: ≥ 13.0	Mean: 60.1–61.9 SD: 7.8–10 (all postmenopausal)	Q1: < 5.8	Q4: > 14.5
Li et al., 2018 [10]	22	–	–	–	32–70 (43–53% postmenopausal)	Q1: < 8.0^c^	Q4: ≥ 15.8^c^
Han et al., 2024 [14]	4.3	Mean: 60 Range: 40–86	5th centile: < 3.0	75th centile: ≥ 5.0	–	–	–

*Q* quartile, *IQR* interquartile range

^a^Reference group

^b^PRL values provided by authors through personal communication (02 April 2024)

^c^Original PRL values: Q1: ≤ 347.8 pmol/l and Q4: ≥ 687 pmol/l (conversion factor: ng/ml × 43.478 = pmol/l)

Sample sizes ranged from 120 to 2,948 participants in cross-sectional studies and 618 to 8,615 in cohort studies. All studies reported men and women separately, some studied only a single sex. Serum PRL was measured by chemiluminescence immunoassay in all studies and presented the unit of measurement in ng/ml by all but one study which reported in pmol/l [10]. Individuals with known pituitary diseases, hyperprolactinemia and medications known to alter circulating PRL levels were excluded by all primary studies. Type 2 diabetes was defined variably in primary studies including self-reported physician’s diagnosis, fasting blood glucose ≥ 126 mg/dL (7.0 mmol/L), two hours plasma glucose during oral glucose tolerance test ≥ 200 mg/dL (11.1 mmol/L), or glycated hemoglobin (HbA_1C_) ≥ 6.5% (48 mmol/mol). The relationship between PRL and type 2 diabetes reported in primary papers were mostly adjusted for confounding factors such as age, body mass index and smoking status, except one study where crude OR was calculated from the reported prevalence of type 2 diabetes [6].

From a total of seven papers selected, five papers included cross-sectional studies comprising 8,720 men (mean age range 51.4–60 years) and 3,429 women (49.5–61.6 years), and four papers included cohort studies comprising 2,948 men (52.1–60.0 years) and 3,203 women (49.2–60.1 years). Most studies used quartiles for categorizing PRL for analysis, except one study which defined PRL below 5th centile as low group and above 75th centile as high group [14]. Some studies used the first quartile whilst other used the fourth quartile as reference group. For clarity of presentation, ORs were inverted where necessary such that the group with highest PRL levels was designated as the reference group. In addition, all units of PRL measurement were converted to ng/ml. Using quartiles in most studies, the PRL cut-off values were variably defined: PRL values in the lowest groups ranged from 3.6 ng/ml to 7.2 ng/ml in men and 4.5 ng/ml to 8 ng/ml in women; and PRL values in the highest groups ranged from 6.9 ng/ml to 13 ng/ml in men and 9.6 ng/ml to 15.8 ng/ml in women. One study defined cut-off for lowest PRL group at 5th centile (3 ng/ml) and highest PRL group at 75th centile (5 ng/ml) [14]. The duration of follow-up amongst cohort studies were between 3.7 and 22 years. The mean or median age range was around 52–60 years in male and 50–62 years in female participants. Two studies included only postmenopausal women whilst one study included a mix of premenopausal and postmenopausal women, and two studies included a mix of those aged above as well as below 50 years (Table 1).

### Meta-analysis

**Fig. 2:**
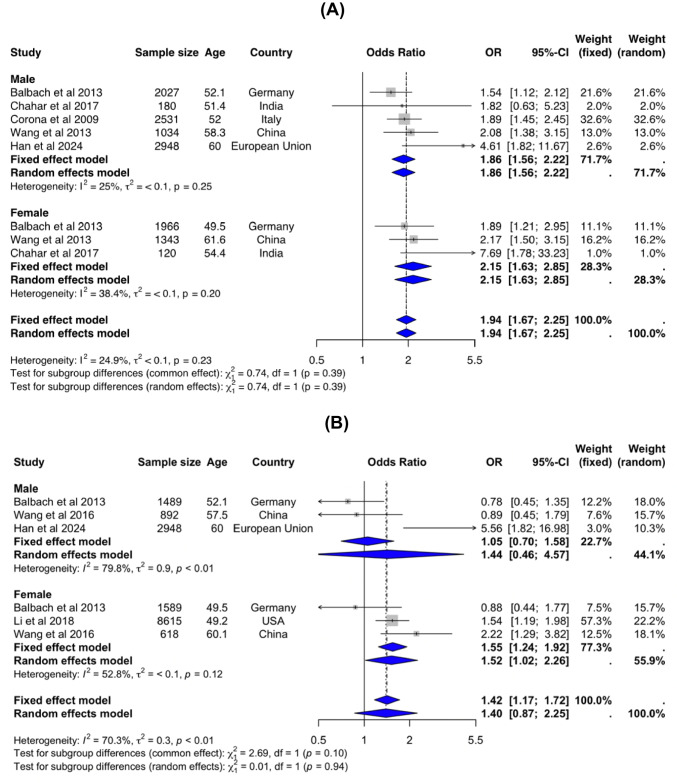
Association between individuals with low levels of PRL and type 2 diabetes reported in cross-sectional studies (**A**), and risk of developing type 2 diabetes amongst individuals with low PRL levels at baseline reported in cohort studies (**B**). Group of individuals with high levels of PRL within physiological range were used as reference (see Table 1 for PRL cut-off values from primary studies). Note: CI, confidence interval; df, degrees of freedom; *P*, probability

In cross-sectional studies, compared to individuals with the highest PRL levels (reference group), those with the lowest PRL levels were associated with higher risk of type 2 diabetes both in men: OR (95%CI) = 1.86 (1.56–2.22) and in women: OR = 2.15 (1.63–2.85) (Fig. 2A) and in women in cohort studies: OR = 1.52 (1.02–2.26) (Fig. 2B). On the other hand, low PRL did not predict type 2 diabetes amongst men in cohort studies: OR = 1.44 (0.46–4.57) (Fig. 2B). Relatively low heterogeneity was observed for cross-sectional studies (*I*^2^ = 25–38.4%), but higher for cohort studies (*I*^2^ = 52.8–79.7%). (Fig. 2A, B).

### Risk of bias within primary studies

There was little evidence of bias due to confounding factors or in the selection of participants except the cohort study by Balbach et al. [7] where almost a quarter of participants were excluded on the grounds that they had metabolic syndrome (Table 2).

**Table 2:** Risk of bias assessed by the Joanna Briggs Institute Critical Appraisal Checklist for cross-sectional studies (**A**) and cohort studies (**B**)

**(A)**												
	**Cross-sectional studies questions**											
**Studies in men**	**1**	**2**	**3**	**4**	**5**	**6**	**7**	**8**	**9**	**10**	**Total** (%)	**Risk of bias**
Corona et al., 2009 [6]	**Y**	**Y**	**Y**	**Y**	**Y**	**N**	**Y**	**Y**	**Y**	**Y**	90	**Low**
Balbach et al., 2013 [7]	**Y**	**Y**	**Y**	**Y**	**Y**	**Y**	**Y**	**Y**	**Y**	**Y**	100	**Low**
Wang et al., 2013 [8]	**Y**	**Y**	**Y**	**Y**	**Y**	**Y**	**Y**	**Y**	**Y**	**N**	90	**Low**
Chahar et al., 2017 [9]	**Y**	**Y**	**Y**	**Y**	**Y**	**Y**	**Y**	**Y**	**Y**	**Y**	100	**Low**
Han et al., 2024 [14]	**Y**	**Y**	**Y**	**Y**	**Y**	**Y**	**Y**	**Y**	**Y**	**Y**	100	**Low**
**Studies in women**												
Balbach et al., 2013 [7]	**Y**	**Y**	**Y**	**Y**	**Y**	**Y**	**Y**	**Y**	**Y**	**Y**	100	**Low**
Wang et al., 2013 [8]	**Y**	**Y**	**Y**	**Y**	**Y**	**Y**	**Y**	**Y**	**Y**	**N**	90	**Low**
Chahar et al., 2017 [9]	**Y**	**Y**	**Y**	**Y**	**Y**	**Y**	**Y**	**Y**	**Y**	**Y**	100	**Low**
**Question 1:** Were the groups comparable other than the presence of disease in cases or the absence of disease in controls?												
**Question 2:** Were cases and controls matched appropriately?												
**Question 3:** Were the same criteria used for identification of cases and controls?												
**Question 4:** Was exposure measured in a standard, valid and reliable way?												
**Question 5:** Was exposure measured in the same way for cases and controls?												
**Question 6:** Were confounding factors identified?												
**Question 7:** Were strategies to deal with confounding factors stated?												
**Question 8:** Were outcomes assessed in a standard, valid and reliable way for cases and controls?												
**Question 9:** Was the exposure period of interest long enough to be meaningful?												
**Question 10:** Was appropriate statistical analysis used												

**(B)**													
	**Cohort studies questions**												
**Studies in men**	**1**	**2**	**3**	**4**	**5**	**6**	**7**	**8**	**9**	**10**	**11**	**Total (%)**	**Risk of bias**
Balbach et al, 2013 [7]	**Y**	**N**	**Y**	**Y**	**Y**	**Y**	**Y**	**N**	**Y**	**Y**	**Y**	81.8	**Low**
Wang et al, 2016 [11]	**Y**	**Y**	**Y**	**Y**	**Y**	**Y**	**Y**	**N**	**Y**	**Y**	**Y**	90.9	**Low**
Han et al, 2024 [14]	**Y**	**Y**	**Y**	**Y**	**Y**	**Y**	**Y**	**N**	**Y**	**Y**	**Y**	90.9	**Low**
**Studies in women**													
Balbach et al, 2013 [7]	**Y**	**N**	**Y**	**Y**	**Y**	**Y**	**Y**	**N**	**Y**	**Y**	**Y**	81.8	**Low**
Li et al, 2018 [10]	**Y**	**Y**	**Y**	**Y**	**N**	**Y**	**Y**	**Y**	**Y**	**Y**	**Y**	90.9	**Low**
Wang et al, 2016 [11]	**Y**	**Y**	**Y**	**Y**	**Y**	**Y**	**Y**	**N**	**Y**	**Y**	**Y**	90.9	**Low**
**Question 1:** Were the two groups similar and recruited from the same population?													
**Question 2:** Were the exposures measured similarly to assign people to both exposed and unexposed groups?													
**Question 3:** Was the exposure measured in a valid and reliable way?													
**Question 4:** Were confounding factors identified?													
**Question 5:** Were strategies to deal with confounding factors stated?													
**Question 6:** Were the groups/participants free of the outcome at the start of the study (or at the moment of exposure)?													
**Question 7:** Were the outcomes measured in a valid and reliable way?													
**Question 8: **Was the follow up time reported and sufficient to be long enough for outcomes to occur?													
**Question 9:** Was follow up complete, and if not, were the reasons to loss to follow up described and explored?													
**Question 10:** Were strategies to address incomplete follow up utilized?													
**Question 11:** Was appropriate statistical analysis used?													

*Y* Yes, *N* No

## Discussion

In this meta-analysis of 28,300 data points, we observe that in cross-sectional studies, individuals with low PRL levels were associated with higher prevalence of type 2 diabetes in both sexes. On the other hand, in cohort studies, low levels of PRL were associated with greater risk of developing type 2 diabetes in women, but not in men.

The discrepancy between cross-sectional and cohort studies in men may be due to factors such as differences in study populations and inconsistency in selection criteria of participants, as well as a paucity of published data. Although the older two cohort studies, one Chinese [8] and the other German [7] showed no relationship between low PRL and incident diabetes in men, our own data in men from eight European countries found that low PRL was a risk factor for incident diabetes [14]. An in-depth examination of these three papers revealed several key factors which could partly explain their differences. Firstly, the cut-offs for defining PRL groups differed between studies. The threshold defined by our group was lowest (3 ng/ml) whilst those from Balbach et al. [7] and Wang et al. [8] were 3.6 ng/ml and 5.7 ng/ml respectively. In men, it is possible that only a very low PRL level could increase the risk of future diabetes. The use of relatively higher PRL cut-offs may suffer from a dilution effect since individuals with lower risk for diabetes are included in the lowest PRL category. The most notable difference identified in the Balbach study [7] to other studies is their exclusion of patients with metabolic syndrome at baseline. This selection may have introduced a bias since many individuals at risk of developing type 2 diabetes may have been omitted. On the other hand, the sample size from the study by Wang et al. [8] was relatively small and a short duration of follow-up, leading to few numbers of incident diabetes. Age differences between studies may also be a crucial factor, particularly premenopausal women since changes in PRL levels are influenced by oestrogens. Furthermore, the occurrence of diabetes increases with age, thus follow-up may require longer in younger populations to have adequate numbers of diabetes. Serum PRL is also known to decline with age [14]. Therefore although the source studies included adjustment for age, there might remain possible residual confounding of the relationship between PRL and type 2 diabetes from very strong association between type 2 diabetes and age, if that association with age is not linear. A previous study found an age effect on the relationship of PRL with glycaemia or insulin sensitivity [21].

Previous studies have found low serum PRL to be related to higher rates of metabolic syndrome [22], and insulin resistance assessed by homeostatic model assessment (HOMA-IR) [23] in men without diabetes; these two conditions are major precursors for developing type 2 diabetes. Other evidence supporting these findings includes a positive correlation between serum PRL (within physiological range) and higher insulin sensitivity and glucose tolerance [21], and with adiponectin levels [24]. Conversely, lower levels of PRL were observed to associate with insulin resistance and adipocyte hypertrophy in the visceral adipose tissue of overweight and obese individuals [25].

We recognize that the effect of low PRL on diabetes, and indeed other metabolic disorders, is difficult to determine probably because the effects are thought to be minor. Nevertheless it is important to elucidate through evidence-based research. One explanation is that the metabolic actions of PRL have been debated but always neglected, probably because they are considered to have minor clinical relevance [26]. The observational studies presented here cannot infer, or exclude causality, and no experimental interventional studies with PRL administration exist in humans. However, studies have shown that PRL levels could be raised by treating castrated men with a combination of pharmacological doses of estrogen (1.5 mg estradiol benzoate once a day for nine days) and gonadotrophin releasing hormone [27]. Animal studies have suggested that PRL involves in regulation of β-cells and insulin action in a dose-dependent manner. Treatment of diabetic rats with low-dose PRL increased β-cell mass and improved hepatic insulin resistance, whilst high-dose PRL treatment led to whole-body insulin resistance [28].

As expected for a meta-analysis, certain limitations were encountered in this study due to differences in subject characteristics and study designs amongst primary studies. Despite including more recent studies in this new meta-analysis, there are still relatively few, and meta-analysis is limited by the high heterogeneity in cohort studies. Given these consistent findings, it would be of interest to conduct interventional studies to examine the effects on health outcomes (including type 2 diabetes) from raising PRL levels, specifically in individuals with low circulating PRL, and to achieve consensus on the definition of hypoprolactinaemia, as proposed in our study [14]. Quick-release formulation of bromocriptine was approved by the US Food and Drug Administration for the treatment of type 2 diabetes as an adjunct to diet and exercise to improve glycemic control in adults in 2009 [29]. This agent could potentially confound the relationship between PRL and type 2 diabetes (only in cross-sectional studies) by reducing the levels of PRL. None of the studies included in this meta-analysis reported the use of bromocriptine treatment for type 2 diabetes, but it is possible that this treatment was not documented.

In conclusion, we have observed that low PRL is associated with type 2 diabetes, but discrepancy between men and women in the relationship within cohort studies deserves further research.

## Data Availability

The data that support the findings of this study are available from the corresponding author upon reasonable request.
